# Role of ARPC2 in Human Gastric Cancer

**DOI:** 10.1155/2017/5432818

**Published:** 2017-06-13

**Authors:** Jun Zhang, Yi Liu, Chang-Jun Yu, Fu Dai, Jie Xiong, Hong-Jun Li, Zheng-Sheng Wu, Rui Ding, Hong Wang

**Affiliations:** ^1^Department of General Surgery, Third Affiliated Hospital (Hefei First People's Hospital) of Anhui Medical University, Hefei, China; ^2^Department of Gastrointestinal Surgery, First Affiliated Hospital of Anhui Medical University, Hefei, China

## Abstract

Gastric cancer continues to be the second most frequent cause of cancer deaths worldwide. However, the exact molecular mechanisms are still unclear. Further research to find potential targets for therapy is critical and urgent. In this study, we found that ARPC2 promoted cell proliferation and invasion in the human cancer cell line MKN-28 using a cell total number assay, MTT (3-(4,5-dimethyl-2-thiazolyl)-2,5-diphenyl-2-H-tetrazolium bromide) assay, cell colony formation assay, migration assay, invasion assay, and wound healing assay. For downstream pathways, CTNND1, EZH2, BCL2L2, CDH2, VIM, and EGFR were upregulated by ARPC2, whereas PTEN, BAK, and CDH1 were downregulated by ARPC2. In a clinical study, we examined the expression of ARPC2 in 110 cases of normal human gastric tissues and 110 cases of human gastric cancer tissues. ARPC2 showed higher expression in gastric cancer tissues than in normal gastric tissues. In the association analysis of 110 gastric cancer tissues, ARPC2 showed significant associations with large tumor size, lymph node invasion, and high tumor stage. In addition, ARPC2-positive patients exhibited lower RFS and OS rates compared with ARPC2-negative patients. We thus identify that ARPC2 plays an aneretic role in human gastric cancer and provided a new target for gastric cancer therapy.

## 1. Introduction

Despite the rapid progression in the therapies used to treat gastric cancer patients, it continues to be the second most common cause of cancer deaths worldwide [1–4]. Patients with gastric cancer usually lack symptoms in the early stages, and efficient early detection methods are limited. Hence, gastric cancers are usually diagnosed only at advanced stages. Combinations of surgery, chemotherapy, and radiotherapy are common treatment options for advanced gastric cancer, but the prognosis is always poor [4, 5]. Many scientists have focused on the molecular mechanisms underlying the development and progression of gastric cancer, but the exact mechanisms remain unclear. Further studies are therefore warranted to find potential therapeutic targets for gastric cancers.

Actin-related protein 2/3 complex subunit 2 (ARPC2) is one of the evolutionarily conserved subunits of actin-related protein 2/3 complex (Arp2/3). The other subunits are ARPC1, ARPC3, ARPC4, and ARPC5 and two actin-related proteins Arp2 and Arp3 [6–9]. In the Arp2/3 complex, ARPC2 subunit holds a central structural position and helps relay both signals and conformational changes [7, 10, 11]. Ghouleh et al. demonstrated that ARPC2 participated in promoting smooth muscle cell migration [6]. Melboucy-Belkhir et al. determined that ARPC2 was regulated by Forkhead box F1 (FOXF1) and might be involved in cell growth of lung fibroblasts [12]. To date, there is no publication that documents the relation between ARPC2 and tumor proliferation or invasion.

In this article, we determined that ARPC2 promotes both proliferation and invasion in the human gastric cancer cell line MKN-28 by using a cell total number assay, MTT assay, cell colony formation assay, migration assay, invasion assay, and wound healing assay. For downstream genes, we found that cancer-promoting genes were upregulated while tumor suppress genes were downregulated by ARPC2. Moreover, in clinical tissues, ARPC2 was associated with tumor size, lymph node invasion, and tumor stage but not with patients' age, gender, or tumor grade. ARPC2 expressed higher levels in gastric cancer tissues than in normal gastric tissues. Furthermore, ARPC2-positive patients exhibited both a lower RFS rate (*P* = 0.009) and a lower OS rate (*P* = 0.030) than did ARPC2-negative patients. As a result, ARPC2 played a destructive role in human gastric cancer cells and can be used as a potential target for the diagnosis and therapy of gastric cancer.

## 2. Materials and Methods

### 2.1. Cell Lines and Cell Culture

Human gastric cancer cell MKN-28 was obtained from ATCC (the American Type Culture Collection) (Rockville, MD, USA). As recommended, MKN-28 was cultured in a humidified incubator at 37°C and 5% CO_2_.

### 2.2. RNA Oligonucleotides and Transfection

Small-interfering RNAs used in this article contained two types of ARPC2-siRNA (designated as siARPC2-1 and siARPC2-2 or siAR-1 and siAR-2) and negative control (designated as siNC). They were obtained from GenePharma (Shanghai, China). Lipofectamin 2000 (Invitrogen, Carlsbad, CA, USA) was used to perform siRNA transfection.

### 2.3. RT-Quantitative PCR (qPCR)

We used RT-qPCR to evaluate the mRNA levels of CTNND1, EZH2, BCL2L2, CDH2, VIM, EGFR, PTEN, BAK, and CDH1, which was performed as described in the previous studies [13]. GAPDH was used as the endogenous control.

### 2.4. Cell Proliferation and Invasion Assays

In this study, the cell total number assay, MTT assay, cell colony formation assay, migration assay, invasion assay, and wound healing assay were carried out to test cell proliferation and invasion. They were all carried out as previously described [13–15].

### 2.5. Western Blot Analysis

Western blot analysis was carried out to test the protein level of ARPC2, which was performed as described previously [13]. *β*-Actin was used as the endogenous control.

### 2.6. Patients and Tissue Samples

In total, 110 paraffin-embedded surgical gastric cancer tissue specimens and 110 paraffin-embedded surgical normal gastric tissue specimens were collected at the First Affiliated Hospital of Anhui Medical University (Hefei, Anhui, China) between 2009 and 2015. All experimental protocols were approved by the Ethical Committee of Anhui Medical University and conform to the principles outlined in the Declaration of Helsinki. The pathohistological diagnosis and grade of the patients was based on the World Health Organization grading systems. This study protocol was approved by the institutional review board. Informed consent forms were obtained from all patients. We followed up the patients with gastric cancer, and the average duration was approximately 5 years.

### 2.7. Immunohistochemistry (IHC)

Immunohistochemistry analysis was carried out as described earlier [16]. In this study, we tested the protein level of ARPC2. The stained sections were reviewed and scored using an Olympus microscope (Olympus America Inc., Melville, NY). ARPC2-positive was designated as more than 20% percent of the tumor cells stained, and ARPC2-negative was designated as 20% percent or less of the tumor cells stained.

### 2.8. Statistical Analyses

For the in vitro experiments, the differences were analyzed using unpaired two-tailed *t*-test. For the experiments involving clinical tissues, the differences were analyzed using Pearson's chi-square test. Patient relapse-free survival (RFS) and overall survival (OS) were analyzed using Kaplan-Meier curves, and the differences were analyzed using the log-rank test. *P* < 0.05 was considered statistically significant.

## 3. Results

### 3.1. ARPC2 Promoted Proliferation of MKN-28 Cells

**Figure 1:**
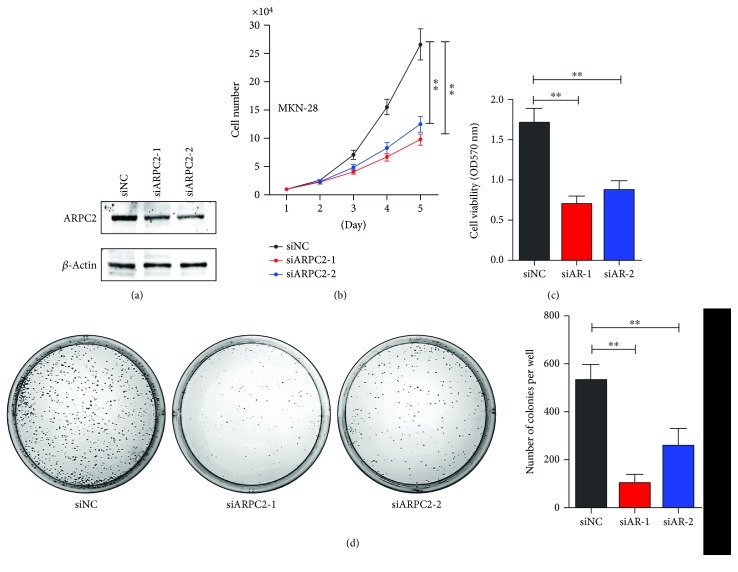
ARPC2 promoted proliferation of MKN-28 cells. MKN-28 cells were transfected with ARPC2-siRNA-1, ARPC2-siRNA-2, or negative control (siNC). (a) Protein level of ARPC2 was evaluated by western blot. (b) Cell total number assay; (c) MTT assay; and (d) cell colony formation assay were performed in MKN-28 cells after transfection. ^∗^*P* < 0.05. ^∗∗^*P* < 0.01.

Human gastric cancer MKN-28 cells were tranfected with ARPC2-siRNA-1, ARPC2-siRNA-2, or negative control (designated as siARPC2-1 and siARPC2-2 or siAR-1, siAR-2, and siNC, resp.).  Figure 1(a) showed that protein level of ARPC2 was dramatically decreased after being tranfected with ARPC2-siRNA-1 and ARPC2-siRNA-2, as determined using western blot. Over a period of 5 days, the cell total number assay showed that the number of MKN-28 cells decreased significantly after being transfected with ARPC2-siRNA-1 and ARPC2-siRNA-2 compared with the negative control (Figure 1(b)). Concordantly, the MTT assay showed that the cell viability of the MKN-28 cells decreased significantly after being transfected with ARPC2-siRNA (Figure 1(c)). Moreover, Figure 1(d) showed that the cell colony formation of MKN-28 cells decreased apparently after being transfected with ARPC2-siRNA-1 and ARPC2-siRNA-2 compared with the negative control. Therefore, ARPC2 promoted the proliferation of human gastric cancer cells.

### 3.2. ARPC2 Promoted Invasion of MKN-28 Cells

**Figure 2:**
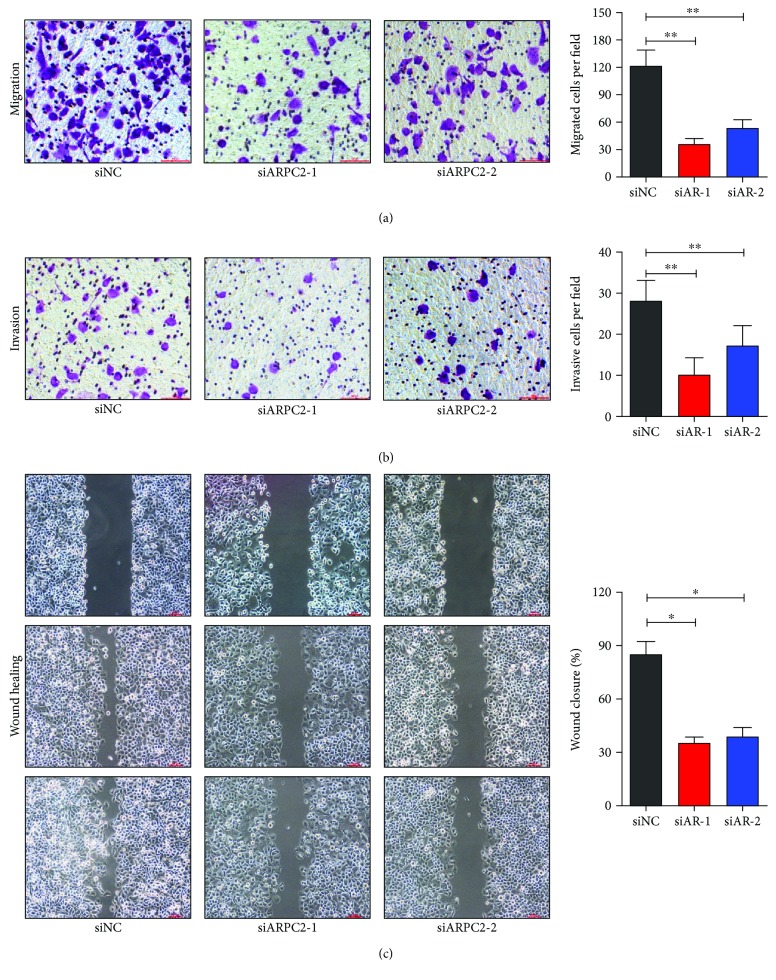
ARPC2 promoted invasion of AGS cells. MKN-28 cells were transfected with ARPC2-siRNA-1, ARPC2-siRNA-2, or negative control. (a) Migration assay; (b) invasion assay; and (c) wound healing assay were performed in MKN-28 cells after transfection. ^∗^*P* < 0.05. ^∗∗^*P* < 0.01.

To further evaluate whether ARPC2 could promote the invasion of MKN-28 cells, migration assay, invasion assay, and wound healing assay were carried out. After transfection with ARPC2-siRNA-1 and ARPC2-siRNA-2, both the migration (Figure 2(a)) and invasion (Figure 2(b)) of MKN-28 cells decreased remarkably compared with the negative control. Additionally, the wound closing of MKN-28 cells was significantly decreased in MKN-28 cells with a decreased expression of ARPC2 (Figure 2(c)). As a result, ARPC2 also promoted the invasion of human gastric cancer cells.

### 3.3. ARPC2 Regulated the Expression of Several Genes

**Figure 3:**
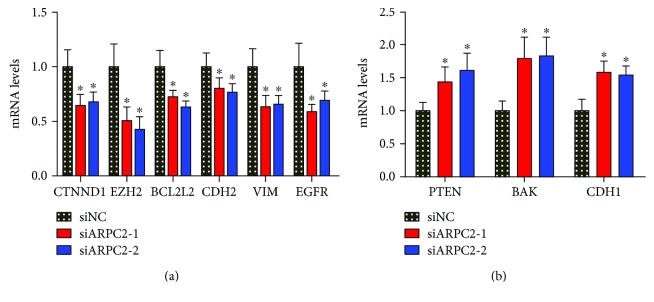
ARPC2 regulated proliferation and invasion-related genes. (a) mRNA levels of CTNND1, EZH2, BCL2L2, CDH2, VIM, and EGFR were decreased after transfected with ARPC2-siRNA-1 and ARPC2-siRNA-2 compared with the negative control using RT-qPCR. (b) mRNA levels of PTEN, BAK, and CDH1 were increased after transfected with ARPC2-siRNA-1 and ARPC2-siRNA-2 compared with the negative control. GAPDH was used as the endogenous control. ^∗^*P* < 0.05. ^∗∗^*P* < 0.01.

In addition, we carried out large-scale RT-qPCR to screen for genes that were regulated by ARPC2. It has been reported previously that CTNND1, EZH2, BCL2L2, CDH2, VIM, and EGFR have positive correlation to proliferation, invasion, and poor prognosis of gastric cancer. As shown in Figure 3(a), the mRNA levels of those genes were clearly decreased after being transfected with ARPC2-siRNA-1 and ARPC2-siRNA-2, which indicated that ARPC2 upregulated them. In contrast, the mRNA levels of tumor suppress genes (PTEN, BAK, and CDH1) were increased significantly after the blocking of ARPC2, which indicated that ARPC2 downregulated them (Figure 3(b)). Therefore, we may conclude that these genes might participate in the role that ARPC2 plays in promoting the cell proliferation and invasion of human gastric cancer cells.

### 3.4. Association of ARPC2 Expression with Clinic-Pathological Features from Patients with Gastric Cancer

**Figure 4:**
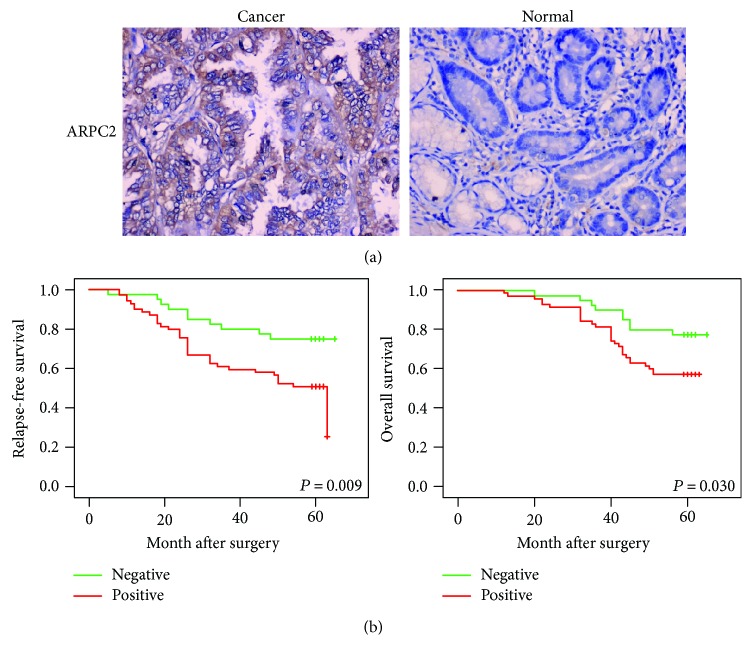
Relapse-free and overall survival curves stratified by ARPC2 expression. (a) Expression of ARPC2 protein in normal gastric tissues and gastric cancer tissues was detected using immunohistochemistry method, and representative pictures are shown. Magnification: 200. (b) Gastric cancer patients with positive expression of ARPC2 were associated with worse relapse-free survival and overall survival.

We collected 110 normal gastric tissues and 110 gastric cancer tissues of archived formalin-fixed paraffin-embedded specimens and detected the protein level of ARPC2 using immunohistochemistry. The positive signal of ARPC2 protein was mainly located in the cytoplasm. As shown in Table 1, in gastric cancer tissues, 40 of the 110 cases negatively expressed ARPC2 and 70 of the 110 cases positively expressed ARPC2; in normal gastric tissues, 70 of the 110 cases negatively expressed ARPC2 and 40 of the 110 cases positively expressed ARPC2. As a result, the percentage of ARPC2-positive tissues in gastric cancer specimens was much higher than that in the normal gastric specimens.  Figure 4(a) shows the typical pictures.

For further study, we associated ARPC2 expression with clinic-pathological features from patients with gastric cancer. The patients' age, gender, tumor size, lymph node invasion, tumor grade, and tumor stage were included. The expression of ARPC2 was markedly higher in patients with a tumor size > 5 cm than in those with a tumor size ≤ 5 cm (*P* = 0.001); higher in tumors with lymph node invasion than in tumors without lymph node invasion (*P* = 0.004); and higher in higher-stage (stages III-IV) tumors than in lower-stage (stages I-II) tumors (*P* = 0.001). However, there was no significant difference between ARPC2 expression and other clinic-pathological features, including patients' age, gender, and tumor grade (Table 2).

### 3.5. Correlation between ARPC2 Expression and Survival of Patients with Gastric Cancer

To evaluate the RFS and OS rates of gastric cancer patients with different levels of ARPC2 expression, Kaplan-Meier analyses were performed in the 110 gastric cancer tissues. Every patient was followed up for more than 5 years. As shown in Figure 4(b), the ARPC2-positive patients exhibited both a lower RFS rate (*P* = 0.009) and a lower OS rate (*P* = 0.030) than did the ARPC2-negative patients.

## 4. Discussion

Herein, we confirmed for the first time that ARPC2 had adverse effects in human gastric cancer, and this was the first report on the role of ARPC2 in human cancers. Combining multiple methods that were used in our study, we drew conclusions that ARPC2 promoted both the cell proliferation and invasion of human gastric cancer cells. In clinical samples, the expression level of APRC2 was much higher in gastric cancer tissues than in normal gastric tissues. Furthermore, the expression of ARPC2 was associated with aggressive behaviors of gastric cancer, including large tumor size, lymph node invasion, high tumor stage, and poor prognosis.

As for recurrent, metastatic, or advanced gastric cancer, the traditional therapeutic methods, including surgery, chemotherapy, and radiotherapy, showed poor curative effects and patient prognoses [4, 12, 17]. Trastuzumab, a monoclonal antibody that targets HER2, is one of a mere few targeted therapies that have been used in human gastric cancer [17, 18]. Trastuzumab could partly prolong survival and improve the quality of life of gastric cancer patients. However, only 15–20% of patients with gastric cancer overexpressed HER2 and could benefit from trastuzumab [17, 19]. Currently, searching for new genes as potential targets for therapy is urgent. In this study, we found that blocking ARPC2 by using the siRNA method can dramatically decrease the cell proliferation and invasion of the human gastric cancer cell line MKN-28. Clinically, an elevated ARPC2 level was associated with a lower survival rate in gastric cancer patients. These results imply that ARPC2 participates in the development of gastric cancer and that function-inhibiting drugs targeted at ARPC2 may be a new approach for its therapy.

As reported previously, Arp2/3 complex is essential for cell motility [11, 20]. As one of the subunits of Arp2/3, ARPC2 also promoted tumor development and progression. For downstream genes, we found that CTNND1, EZH2, BCL2L2, CDH2, VIM, and EGFR were upregulated by ARPC2 and that PTEN, BAK, and CDH1 were downregulated by ARPC2. In previous studies, CTNND1 was documented to promote many types of human cancers, including hepatocellular carcinoma and lung cancer [21, 22]. EZH2 was demonstrated to be oncogenic in esophageal cancer, lung cancer, and breast cancer, among others [23–25]. Moreover, Liu et al. and Chen et al. proved that EZH2 promoted the progression and invasion of human gastric cancer [26, 27]. BCL2L2 was determined to promote tumorigenicity and invasion in human glioblastoma, non-small-cell lung cancer, and colon cancer [28–30]. CDH2 was reported to be related to the epithelial-mesenchymal transition (EMT) in non-small-cell lung cancer [31]. Vimentin (VIM) is a famous EMT marker that promotes tumor invasion and drug resistance in ovarian cancer, colon cancer, and gastric cancer [32–34]. EGFR is also a famous oncogene in ovarian cancer, breast cancer, and gastric cancer [35–37]. Moreover, PTEN is a well-studied gene that inhibits tumor growth and invasion in nearly all types of human cancers, including gastric cancer, breast cancer, and lung cancer [38–41]. BAK was demonstrated to promote tumor apoptosis and chemosensitivity to chemotherapeutic drugs in non-small-cell lung cancer and gastric cancer [42, 43]. CHD1 was also reported to be a tumor suppressor gene in many human cancers [44, 45]. These publications all supported our result. Thus, CTNND1, EZH2, BCL2L2, CDH2, VIM, EGFR, PTEN, BAK, and CDH1 all contributed to the bad role of ARPC2 in human gastric cancer.

In this study, we reported for the first time the destructive role of ARPC2 in human gastric cancer cells. Both in vitro and clinical studies were performed. A high expression of ARPC2 was associated with both poor RFS and OS rates in gastric cancer patients. Therefore, we propose ARPC2 as a new potential biomarker and therapeutic target for patients with gastric cancer.

## Figures and Tables

**Table 1:** Expression of ARPC2 in gastric cancer and normal tissues.

Group		ARPC2 expression	
	*n*	Negative, *n* (%)	Positive, *n* (%)
Cancer	110	40 (36.4)	70 (63.6)^∗^
Normal	110	70 (63.6)	40 (36.4)

Note: ^∗^*P* < 0.001.

**Table 2:** Correlation of ARPC2 expression with clinicopathological parameters from gastric cancer patients.

Parameter	*n*	ARPC2 expression	
		Positive, *n* (%)	*P* value
Age (years)			
≤60	57	34 (59.6)	0.367
>60	53	36 (67.9)	
Gender
Male	60	34 (56.7)	0.096
Female	50	36 (72.0)	
Tumor size (cm)			
≤5	73	38 (52.1)	0.001
>5	37	32 (86.5)	
Lymph node metastasis			
No	34	15 (44.1)	0.004
Yes	76	55 (72.4)	
Grade			
I	11	8 (72.7)	0.638
II	71	43 (60.6)	
III	28	19 (67.9)	
Stage			
I-II	57	25 (43.9)	0.001
III-IV	53	45 (84.9)	
